# Refining taxonomic identification of microalgae through molecular and genetic evolution: a case study of *Prorocentrum lima* and *Prorocentrum arenarium*

**DOI:** 10.1128/spectrum.02367-23

**Published:** 2024-04-04

**Authors:** Danlin Zheng, Ligong Zou, Jian Zou, Qun Li, Songhui Lu

**Affiliations:** 1 College of Life Science and Technology, and Southern Marine Science and Engineering Guangdong Laboratory (Zhuhai), Jinan University, Guangzhou, China; Tecnologico de Costa Rica, Cartago, Costa Rica

**Keywords:** species delimitation methods, phylogeny, orthologous genes, evolution, Ka/Ks analysis

## Abstract

Species delimitation based on lineage definition has become increasingly popular. However, these methods have been limited, especially for species that lack genomic data and are morphologically similar. The trickiest part for the species identification is that the interspecific and intraspecific boundaries are vague. Taking *Prorocentrum* (Dinophyta) as an example, analysis of cell morphology, growth, and toxin synthesis in both species of *P. lima* and *P. arenarium* does not provide a reliable basis for species delineation. However, through phylogenetic and genetic distance analyses of their ITS and LSU sequences, establishment of evolutionary tree based on orthologous gene sequences, and combining the results of automatic barcode gap discovery and Poisson tree processes models, it was sustained that *P. arenarium* does not belong to the *P. lima* complex and should be considered as an independent species. Interspecies genetic evolution analysis revealed that *P. lima* and *P. arenarium* may contribute to evolutionary direction that favors combating reverse environmental factors. In *P. lima*, viral invasion may be one of the reasons for its large genome size. In the study, *P. lima* complex has been selected as an example to enhance the taxonomic identification of microalgae through molecular and genetic evolution, offering valuable insights into refining taxonomic identification and promoting microbial biodiversity research in other species.

**IMPORTANCE:**

Microalgae, especially the species known as *Prorocentrum*, have received significant attention due to their ability to trigger harmful algal blooms and produce toxins. However, the boundaries between species and within species are ambiguous. Clear and comprehensive species delineation indicates that *Prorocentrum arenarium* should be considered as an independent species, separate from the *Prorocentrum lima* complex. Improving the classification and identification of microalgae through molecular and genetic evolution will provide reference points for other cryptic species. *Prorocentrum* occupy multiple ecological niches in marine environments, and studying their evolutionary direction contributes to understanding their ecological adaptations and community succession.

## INTRODUCTION

The identification of species remains a prominent and demanding research pursuit. Because the boundaries between species are still indistinct and there is a lack of defining standards, it is estimated that there are millions of species that have yet to be described (1). Algae are important aquatic organisms for understanding ecosystem processes, conservation, and water quality. Accurate identification of algae is essential for studies of algal genetics, physiology, ecology, and applied phycology, especially for bioassessment (2). Precise taxonomic identification provides consistency and transferability for ecological inferences (3).

The marine microalgae *Prorocentrum*, which encompasses around 84 species (4), is one of the genus from Dinophyceae. This genus has been subject to continuous revisions owing to the ongoing discovery and description of novel species (5–8). Unfortunately, due to the relatively simple morphology of species, *Prorocentrum* are still ambiguous and controversies in the inter- and intraspecific boundary. *Prorocentrum* has gained increasing attention among researchers because some of species from this genus produce okadaic acid (OA) and its related derivatives (dinophysitoxins, DTXs) (9). *Prorocentrum lima* has been shown to produce phycotoxins that cause “diarrhetic shellfish poisoning,” a type of severe gastrointestinal disease in seafood consumers (10). Consumption of shellfish contaminated by DSTs (diarrheic shellfish toxins) may cause stomach spasms, diarrhea, vomiting, and other symptoms (11).

The genus *Prorocentrum* was originally described by Ehrenberg in 1834, with *Prorocentrum micans* Ehrenberg serving as its type species (12, 13). Dodge (14) conducted a comprehensive taxonomic revision of *Prorocentrum* species, including *P. lima* (14). *P. lima* cells exhibit an oblong-to-ovate shape, with a broad middle region and narrowing toward the anterior end, achieving their maximum width below the cell’s center (15). These cells are encased by two cellulose thecal plates known as valves (16). The surface of these valves is smooth, and both valves possess pores, except in the central cell area. Additionally, a row of marginal pores is present at the cell’s periphery (15). The size and valve shape of *P. lima* are variable, but the threshold value is uncertain (17), with ellipsoid, ovoid, or round shapes, with ovoid shapes being the most common (14, 18–20). In addition, there are differences in the shape and number of valve pores and marginal pores, the megacytic growth zone (as an intercalary band), and the arrangement of platelets within *P. lima*, and their variability hinders species identification within the so-called “*Prorocentrum lima* complex” (8, 15, 21–23).

Nagahama et al. proposed that the morphology of *P. arenarium* falls within the range of morphological variation observed in *P. lima* and considered *P. arenarium* as a synonym of *P. lima* (21), but Nascimento et al. considered it as a different species as *P. arenarium* having a wider mid-cell area and a round or wide-ovoid shape (24). Chomérat et al. showed that the proposal of this synonym has not been universally accepted by all authors (8). Arteaga-Sogamoso et al. (22) still considered *P. arenarium* as part of the *P. lima* complex (22). This issue remains controversial; it is necessary to develop a combination of other methods for research based on previous species definition. Consequently, due to the plasticity of certain characteristics and the presence of cryptic species, the taxonomy of the *Prorocentrum* genus remains complex and contentious. Identifying species boundaries within this genus remains challenging to this day.

Access to genomic data makes species delimitation increasingly feasible (25). Typical dinoflagellates genome is roughly 1–80 times that of the human haploid genome (26). Because of the large genome of *Prorocentrum* being difficult to sequence (27), there is a lack of available genomic data for species delimitation. Currently, there is limited information for defining *Prorocentrum* species. The available methods include morphology, LSU, and internal transcribed spacer region (ITS) (22). However, these methods lead to controversial species delimitation results. The most reliable method for accurately delineating cryptic species is to use independent methods that allow for cross-validation between different species delimitation methods (SDMs) (28), alternative sources of information (including morphology, biogeography, and behavioral characteristics), known as integrative taxonomy (29). This molecular-assisted approach is now ubiquitous in contemporary algal species descriptions (30), having uncovered numerous hidden species within morphologically defined taxa (31–33), and is the recommended approach for identifying and creating a more natural classification of *Prorocentrum*. Some species lack standards for delimitation. These standards include morphology, reproductive patterns, metabolites, ecological niches, and genomes. To delineate these species, we propose a multimodal approach. This approach includes morphological and ultrastructural observations, phylogenetic analysis (ITS, LSU), sequence alignment of orthologous genes, and DNA-based species delimitation [using the automatic barcode gap discovery (ABGD) method and Poisson tree processes (PTP) model] (34). Multiple markers application significantly improves the accuracy of species delimitation (35), and the consistency of results obtained from multiple molecular SDMs may increase the reliability of the results (33).

The phenotype of species is an important predictor of the distribution and abundance of organisms (36). It is particularly important if species are correctly identified. The taxonomic identification of algae presents a valuable opportunity to comprehend systematic entities and to establish connections with evolutionary and ecological processes (37), as the evolution of traits confers physiological and morphological adaptations that facilitate survival in a diverse range of environmental conditions (38). This widespread diversity is due to their evolutionary adaptation to diverse ecological niches (39). Dinoflagellates possess a larger genome size and many atypical features (26, 40), that is, cells carry an excess of genes and bear significant adaptation costs [e.g., the Black Queen hypothesis, (41)]; this contradicts current evolutionary theories (42). Therefore, dinoflagellates are challenging to categorize due to the difficulty in obtaining their genomes, and our understanding of their evolutionary status remains limited. Understanding the evolutionary status of a species can provide more references for its definition. This study uses dinoflagellates as an example, as they have significant ecological and evolutionary importance (43). Understanding the taxonomy and ecological evolution of *Prorocentrum* can provide vital information for the prediction and management of toxic algal blooms. The first gene evolution analysis of *P. lima* and *P. arenarium* was conducted to explain their evolutionary directions, providing more references in the interspecific boundary determination.

## RESULTS

### Morphological observations and metabolite analysis

**Fig 1:**
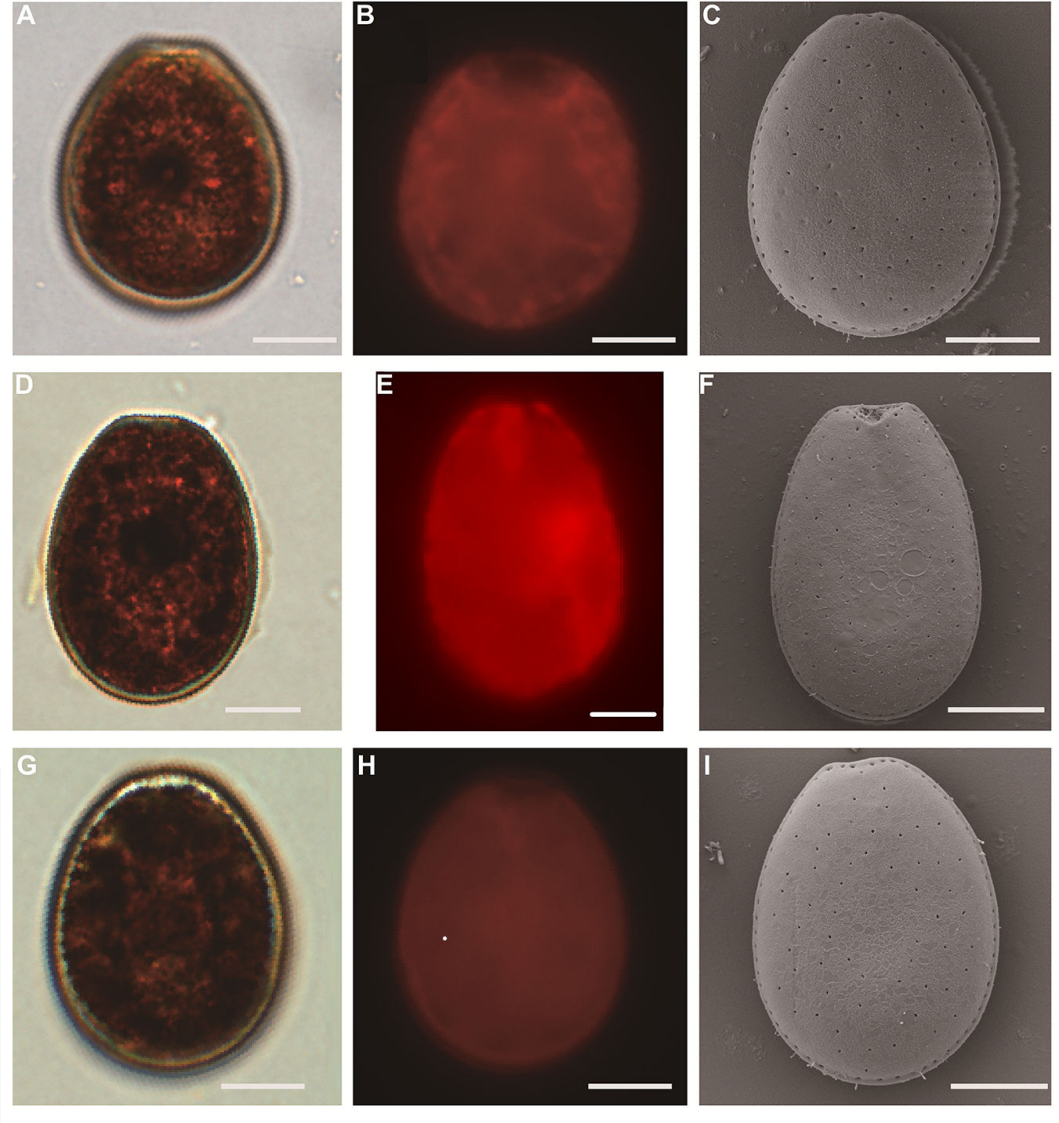
Cell morphology of HN231, XS336, and 3XS36 (A–I). (**A**) Light microscopy (LM), a cell showing pyrenoid, (**B**) fluorescence microscope (FM), epifluorescence image of chloroplasts, (**C**) SEM in HN231; (**D**) LM, a cell showing pyrenoid, (**E**) FM, epifluorescence image of chloroplasts, (**F**) SEM in XS336; (**G**) LM, a cell showing pyrenoid, (**H**) FM, epifluorescence image of chloroplasts, (**I**) SEM in 3XS36. Scale bars, 10 µm.

The study revealed that the cells of these strains were symmetrical, ovate-oblong to ovate in shape, with a narrow anterior end and broad middle region that reached maximum width below the cell’s center (Fig. 1). These were solitary, armored, and photosynthetic cells contained green to golden-brown chloroplasts located at the lateral and posterior regions. Through scanning electron microscopy (SEM) (Fig. 1), the researchers observed that the thecal was smooth and had randomly distributed pores on the lateral plates except for the central region. A row of evenly sized but unevenly spaced marginal pores was found at the periphery of both lateral plates. The valve edges and marginal pores were also smooth. These features are common to both *P. arenarium* and *P. lima* complex, making it difficult to differentiate between the two species based solely on morphological observations. Compared with *P. Lima*, *P. arenarium* exhibits a round or wide-ovoid shape, as indicated by the findings of this study (24). The length-to-width (L/W) ratios of HN231, XS336, and 3XS36 showed significant differences (Fig. S1), and HN231 has maximum L/W ratios, with wide oval shape (Fig. 1), but HN231 L/W ratio variation was within the range of *P. lima* (1.03 and 2.05) (21).

We analyzed the growth and toxin production of closely related species in an attempt to provide a basis for taxonomic identification. During the first 10 days of cultivation, strains (HN231, XS336, 3XS36) all reached their maximum growth rate and completed the exponential growth phase (Fig. S2A and B). The parameters of growth vary from one strain of algae to another (Table S2). The toxicity (content of multiple toxins) levels were measured at various stages of cell growth (Fig. S3). As the culture time increased, the concentrations of OA and DTXs (in both free and total forms) also increased in cells of different strains, reaching a peak during the stable growth period (Fig. S3C, D, G, H, K, and L). All strains produced OA in this study, with similar trends in the rates of production of free or total OA content (Fig. S3C and D). The strain HN231 produced OA, and the detection results for DTX1 and DTX2 were below the detection limit or at extremely low levels. It is summarized that one strain of *P. lima*, XS336, produced OA and DTX1, while another strain, 3XS36, generated OA and DTX2 isomer. However, the total amount of OA varied significantly among the three strains (Table S3), with HN231 showing the highest total OA content (ranging from 5.90 to 33.89 pg/cell), while XS336 and 3XS36 had similar, but lower total OA content than HN231. Total/free OA and total/free DTX content showed a high degree of linearity (Fig. S4). These results demonstrate that the production rates, type, and levels of OA and DTXs vary among different strains (44).

### Molecular phylogenetic analysis and species delimitation

**Fig 2:**
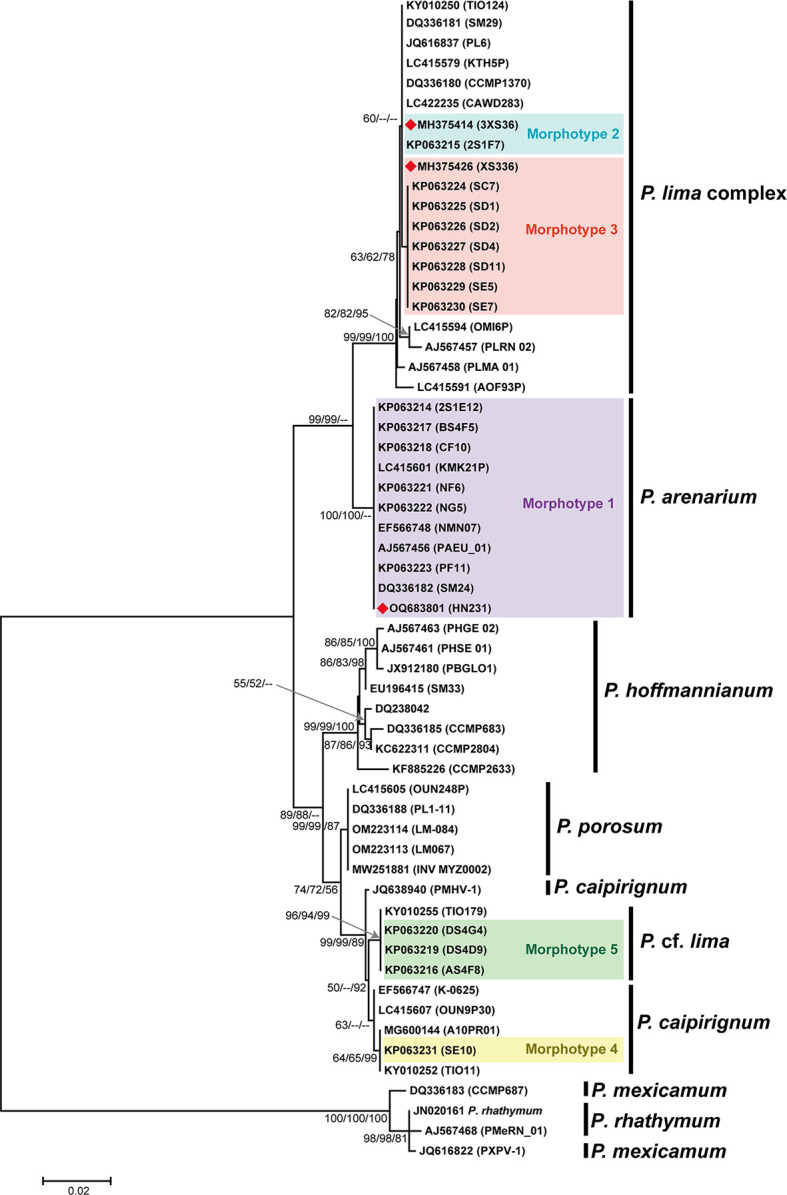
The phylogenetic trees of the *Prorocentrum* based on LSU involved 58 nucleotide sequences consisted of 970 positions. The phylogenetic tree was constructed using three methods [Bayesian inference (BI), neighbor joining (NJ), and maximum likelihood (ML)] with bootstrap values reported as NJ (bt)/ML (bt)/BI (bt) (*P* bootstrap value). Nodal support represents NJ/ML/BI bootstrap values and posterior probability (>50). Morphotypes 1–5 proposed by Zhang et al. (17).

The LSU phylogeny revealed that the *Prorocentrum* species/phylotypes in the *P. lima* complex and *P. arenarium* were closely related, while HN231 [*P. arenarium*, formerly *P. lima* morphotype 1 (17)] was placed in a distinct branch, supported by high bootstrap and probability values (100/100/--) (Fig. 2). The mean *P*-distances between *P. arenarium* and *P. lima* complex/*P. porosum*/*P. hoffmannianum* were 0.022, 0.040, and 0.048, respectively, higher than the genetic distance between *P. porosum* and *P*. cf. *lima*/*P. caipirignum* (0.014 and 0.013) (Table S4).

**Fig 3:**
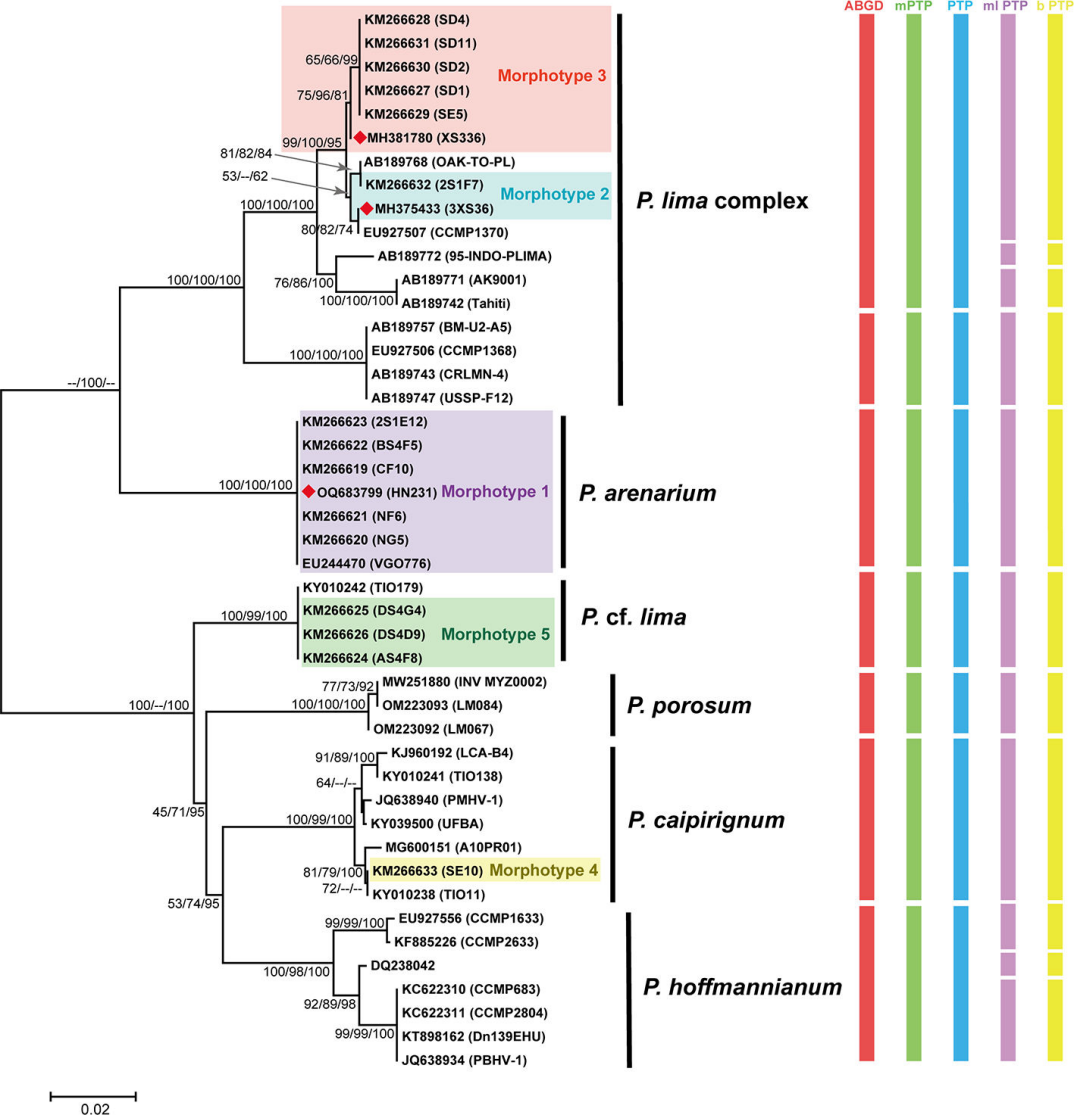
The phylogenetic trees of the *Prorocentrum* based on ITS involved 45 nucleotide sequences consisted of 656 positions. The phylogenetic tree was constructed using three methods [Bayesian inference (BI), neighbor joining (NJ), and maximum likelihood (ML)] with bootstrap values reported as NJ (bt)/ML (bt)/BI (bt) (*P* bootstrap value). Nodal support represents NJ/ML/BI bootstrap values and posterior probability (>50). Morphotypes 1–5 proposed by Zhang et al. (17). The rectangles indicate clustering by various methods of species delimitation: ABGD, mPTP, PTP, ml PTP, b PTP.

The uncorrected genetic distance (*P*) based on ITS and LSU regional sequence calculations showed that the genetic distances within different *Prorocentrum* species were *P* < 0.029 and 0.009, respectively, while the interspecies differences showed *P* values >0.061 and 0.006, respectively. Phylogenetic tree and genetic distance analysis showed that the ITS sequence was more sensitive than the LSU sequence in distinguishing between species (21). Regarding the ITS phylogenetic analysis, HN231 (*P. arenarium*, formerly *P. lima* morphotype 1) and *P. lima* complex were placed in a distinct branch, supported by high bootstrap and probability values (100/100/100) (Fig. 3). The *P*-distances observed between *P. arenarium* and its closest species/phylotypes (0.099 and 0.139 for the *P. lima* complex and *P*. cf. *lima*, respectively) and the corresponding values between *P*. cf. *lima* and *P. porosum*/*P. hoffmannianum* are 0.061 and 0.071, respectively (Table S5). Phylogenetic analysis of the ITS and LSU gene trees shows that HN231 and the cell representatives of *P. arenarium*, VGO776 (ITS) and PAEU_01 (LSU), are in the same branch.

To evaluate whether *P. arenarium* is a different species compared to *P. lima* complex, species delimitation was performed using ABGD, mPTP, PTP, b PTP, and ml PTP methods. These methods involve the construction of phylogenetic trees obtained through the application of both Mega and Bayesian approaches, utilizing various program models for computations to ensure the acquisition of robust results. The results of the ABGD, mPTP, and PTP methods were consistent, identifying seven molecular operational taxonomic units (MOTUs) at the species level in *Prorocentrum*, which were the same as the species identified based on the phylogenetic analysis (Fig. 3). The species delimitation results of b PTP and ml PTP were more detailed, identifying 11 MOTUs (Fig. 3). All delimitation methods supported that *P. arenarium* studied was distinct to *P. lima* complex and belonged to an independent species.

### Synonymous and nonsynonymous sites between the orthologous genes

**Fig 4:**
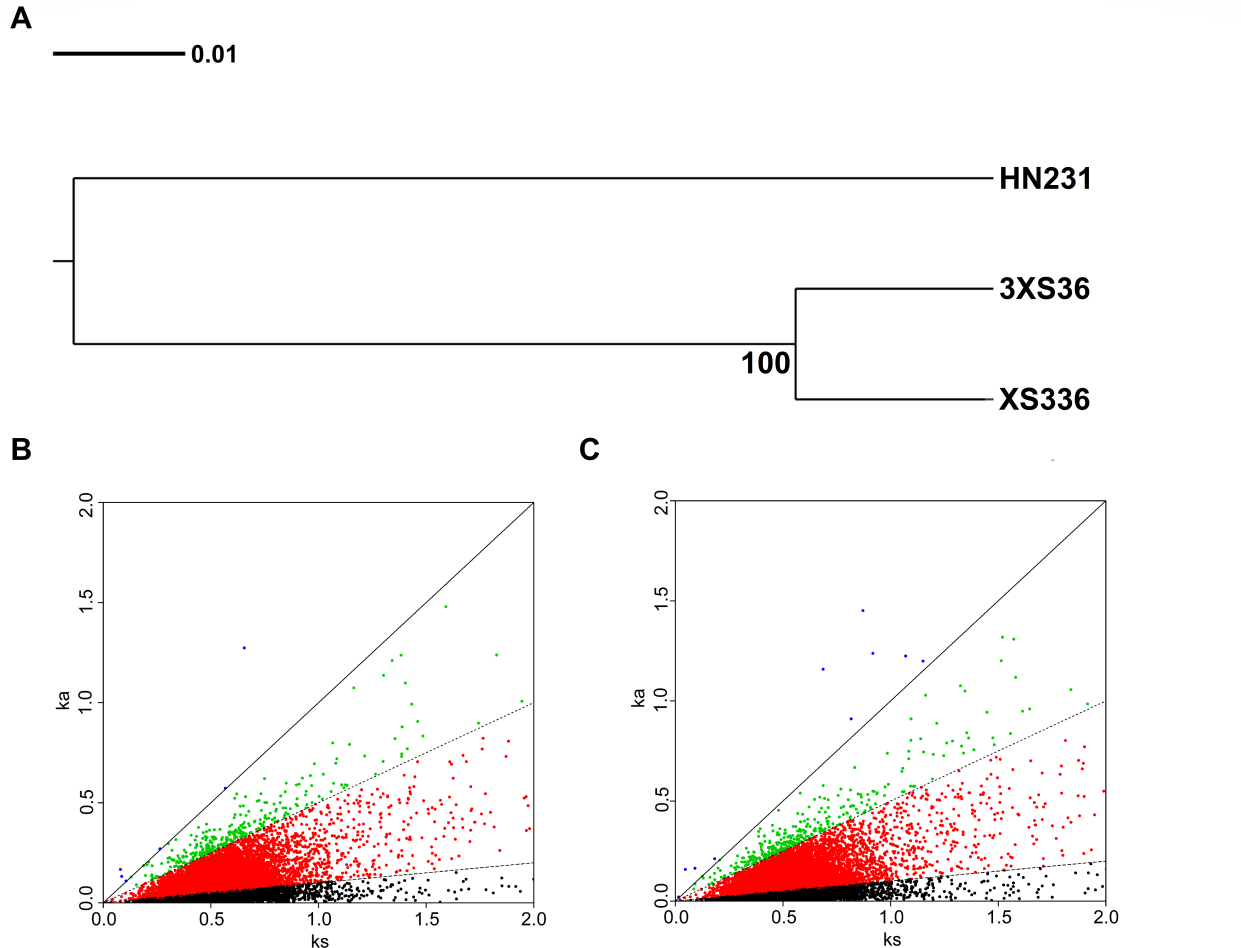
(**A**) Phylogeny of HN231, XS336, and 3XS36 aligned fragments was based on the protein sequences of orthologous genes and constructed by the neighbor-joining method. (**B**) Distribution of Ka and Ks between HN231 and XS336, (**C**) between HN231 and 3XS36. Sequences with Ka/Ks >1 fall above the solid line; while sequences with Ka/Ks between 0.5 and 1 fall between the solid and dashed lines.

This study obtained a total of 27,632 single-copy orthologous genes among species (Table S6). Using 27,632 conserved single-copy orthologous genes, a neighbor-joining (NJ) molecular evolutionary tree was constructed using MEGA based on the differences in protein sequence or structural relationships (Fig. 4A). The length of the branches of the evolutionary tree reflects the evolutionary distance between proteins. It confirmed the previous analysis that XS336 and 3XS36 are in the same branch of the tree and have a high confidence level (100), while XS336 and 3XS36, HN231 are in two different branches and have a distant genetic distance (Fig. 4A). The level of sequence divergence of the orthologous gene sequences further supports that XS336/3XS36, HN231 are different species.

**Fig 5:**
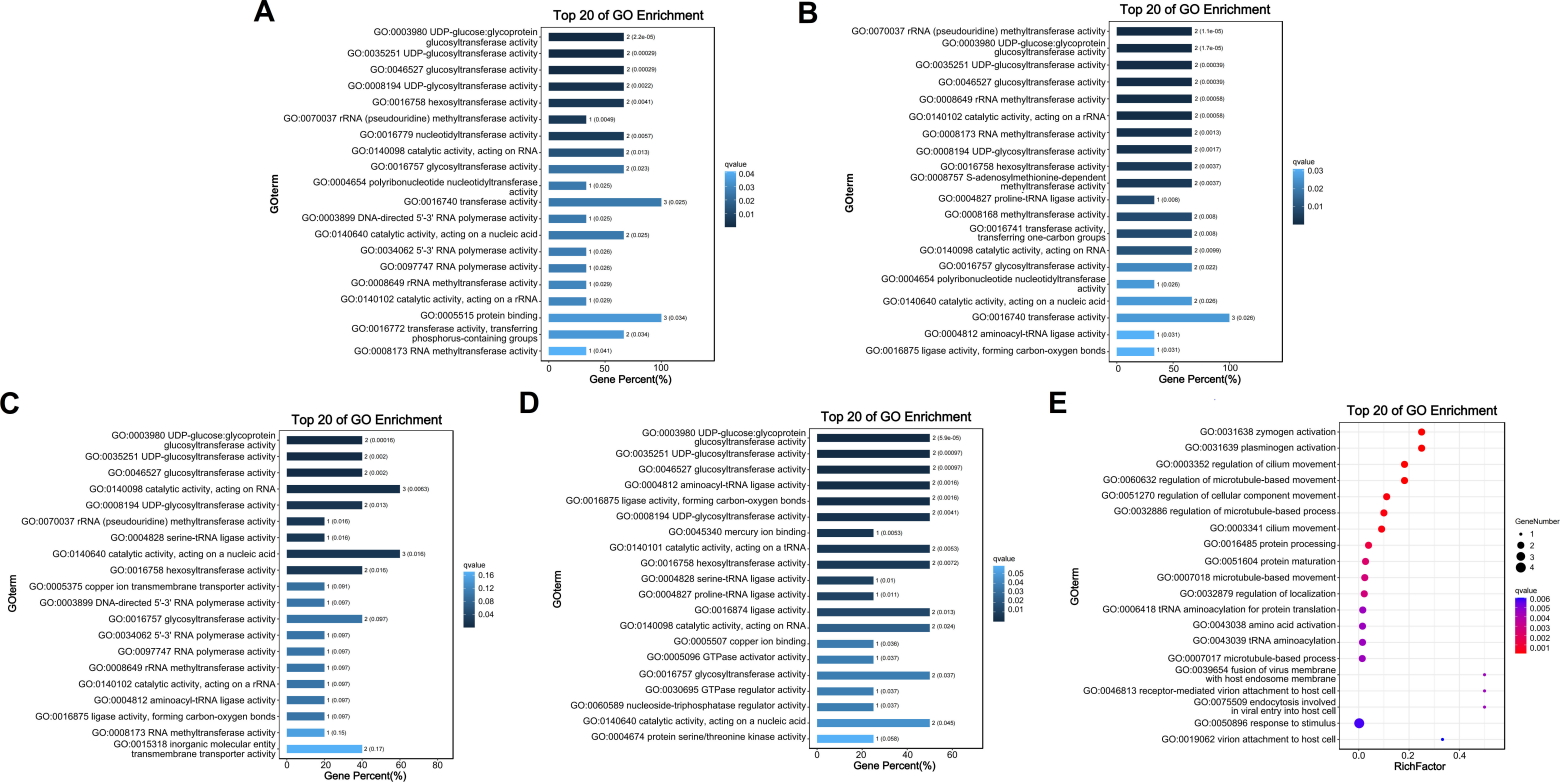
Gene ontology functional enrichment analysis of positive selection genes through molecular function between strains. (**A**) HN231 vs (**B**) XS336; (**C**) HN231 vs (**D**) 3XS36. (**E**) Gene ontology functional enrichment analysis of positive selection genes through biological process of 3XS36.

Out of the 27,631 pairs of orthologous genes between HN231 and XS336, Ka, Ks, and Ka/Ks ratios could be calculated for 27,610 pairs of them (Table S7). The average values of Ka, Ks, and Ka/Ks ratios for these 27,610 sequence pairs were 0.0768, 0.5199, and 0.1418, respectively, which were similar to the average Ka, Ks, and Ka/Ks ratios between HN231 and 3XS36 (0.0777, 0.5219, and 0.1423) (Table S8). Fisher’s exact test reports that nearly 99.4% of the genes have statistical significance (*P* < 0.01). We conducted gene ontology (GO) analysis on positive selection genes (Fig. S5). Between HN231 and XS336, six pairs of orthologous genes have Ka/Ks ratios >1 (Fig. 4B), indicating strong positive selection acting on these genes, and another 1.08% (298 genes) of the genes have Ka/Ks ratios between 0.5 and 1, indicating weaker purifying selection (Table S7). For the pairs of orthologous genes between HN231 and 3XS36, there are 10 pairs of them with Ka/Ks ratios >1 (Fig. 4C), and another 1.20% (332 genes) of the genes have Ka/Ks ratios between 0.5 and 1 (Table S8). The genes undergoing strong positive selection in *P. arenarium* and *P. lima* are primarily involved in energy metabolism, specifically oxidative phosphorylation and photosynthesis. They also include genes related to ubiquitin-mediated proteolysis and UDP-glycosyltransferase activity (Fig. 5A through D). In the Ka/Ks analysis of orthologous genes for HN231 and 3XS36, a portion of the positively selected genes (PSGs) for 3XS36 belongs to responses to stimulus (GO: 0050896), fusion of virus membrane with host endosome membrane (GO: 0039654), virion attachment to host cell (GO: 0019062), receptor-mediated virion attachment to host cell (GO: 0046813), and endocytosis involved in viral entry into host cell (GO: 0075509) (Fig. 5E).

## DISCUSSION

### Polyphasic species delimitation

Cryptic diversity, characterized by the presence of multiple distinct species mistakenly classified as a single entity due to morphological similarities, is considered a potentially significant factor that could impact future conservation decisions (45). Marine algae exhibit a wide range of morphological diversity. Due to their morphological plasticity and lack of useful characteristics, morphological species identification of algae presents many problems, especially for morphologically simple algae (46). Cryptic diversity has been reported in *P. lima* and *P. fukuyoi* (8, 17), but it is unclear if this diversity is linked to geographic origins. For this reason, the use of molecular tools to delimit species in algae has become a common practice, leading to the discovery of a large number of cryptic species (47, 48). rRNA and ITS have been widely used in taxonomic studies because the phylogenetic relationships based on these markers are largely consistent not only with traditional taxonomic classifications but also used to differentiate cryptic/pseudo-cryptic species (37, 38).

There is still ambiguity in the species boundaries of *Prorocentrum*. The most recent molecular phylogenetic analysis of *Prorocentrum* from the eastern Caribbean Sea based on LSU rDNA sequences clusters *P. mexicanum* closely together with *P. rhathymum* (Fig. 2). Currently, the species boundaries of *P. rhathymum* and *P. mexicanum* are still unclear (8, 49). There are still controversies regarding the species delimitation of certain algal species, such as certain *Prorocentrum* species. Recently, *P. maculosum* was no longer considered a synonym of *P. hoffmannianum* (7). Within the genus *Prorocentrum*, there exist several similar yet unresolved taxa provisionally assigned to the *Prorocentrum lima* species complex as suggested by Aligizaki (23). Classification at the species level, however, does not always align between morphological and molecular genetic criteria (49). The considerable phenotypic variability observed defined *Prorocentrum* species has often led to misidentification (49). Taxonomically, the *P. lima* complex poses challenges due to morphological plasticity and the absence of genomes, making species definition particularly problematic. Early molecular studies involving 18S rDNA gene sequences (50) indicated a striking similarity between *P. lima* and *P. arenarium*, with just 11 nucleotide substitutions between them and several shared characteristics. This congruence led to the classification of *P. arenarium* as a round *P. lima* morphotype, a classification later supported by Zhang et al. (17). *P. arenarium* has diverse geographic distributions and ecological niche, and it is difficult to define species according to ecological niche. We used an integrated approach to the analysis, using the *P. lima* complex as an example to define interspecific boundary. In this study, we used a species delimitation polyphasic approach to delimit its species.

Accurately identifying the ecological, distributional, and toxicological characteristics of a species is a fundamental requirement for species identification (51). *P. lima* can produce toxins, including OA, DTX1, DTX2, and their diol esters (9). *P. arenarium* strain was toxicogenic including OA (52); however, there is relatively little research on its toxin production. Based on the growth status and toxin synthesis, it is difficult to define the interspecific boundary of the *P. lima* complex and its closely related species *P. arenarium*. We recommend using multimodal methods to delineate species, including morphological and ultrastructural observations, phylogenetic analysis (ITS, LSU), sequence alignment of orthologous genes, and DNA-based species delimitation (using the ABGD method and PTP model).

Ribosomal RNA genes (LSU, SSU) are widely used to assign putative microalgae species. However, ITS rDNA differentiates faster during species formation, provides better resolution between recently diverged taxa, and has been proposed as an appropriate locus for DNA barcoding of dinoflagellate species (53). The genetic distance of ITS is significantly greater than 0.04, which is a threshold for describing the boundaries of most free-living dinoflagellate species (53). By comparing the results revealed by ITS and LSU rDNA phylogenetic trees, *P. arenarium* and *P. lima* complex belong to two branches with high support, and the genetic distance of ITS is 0.099 (>0.04), supporting two branches have distinct species-level divergence (Fig. 2 and 3). By drawing an evolutionary tree based on 27,632 single-copy orthologous genes, we could more accurately verify this conclusion (Fig. 4A). The interspecific genetic distance of *P. arenarium* branch is 0.000, including strains from China (2S1E12, BS4F5, CF10, NF6, NG5, and HN231), Japan (KMK21P), Malaysia (NMN07), the south west Indian ocean (PAEU_01), Mexico (PF11), and Australia (SM24) (Fig. 2).

Recently, there has been a growing popularity in methods used to identify new species or test species hypotheses, such as ABGD, GMYC, and PTP. These species delimitation methods have been applied to many species, including *Chlorophyta* (34), *Polysiphonia scopulorum* (54), *Kumanoa* (55), *Hypnea cornuta* complex (56), *Pinnularia* (57), *Eubrownia*, *Spongiococcum*, and *Chlorococcum* (58). Each species delimitation method represents only a preliminary hypothesis about species validity, and confirmation is needed through other features. In this study, based on ABGD, PTP, and mPTP, the species delimitation results were consistent, with seven MOTUs identified, which were similar to the results obtained by phylogenetic tree topology and genetic distance. The only difference was in the delimitation of the *P. lima* complex, which needs further investigation on whether the branch (BM-U2-A5, CCMP1368, CRLMN-4, and USSP-F12) still belongs to the *P. lima* complex. The b PTP and ml PTP methods were used to delimit *Prorocentrum* species, and the results showed strong convergence and sensitivity, with 11 MOTUs identified (Fig. 2). All species delimitation methods in this study identified *P. arenarium* as an independent species, and ABGD, mPTP, and PTP more accurately reflected the classification of *Prorocentrum* species. Therefore, based on phylogenetic analysis (ITS, LSU) and species delimitation methods (ABGD, PTP model), it is supported that *P. arenarium* should be considered a new species rather than just a part of the *P. lima* complex. Here, we argue that interspecific boundaries are adequately defined by a combination of methods, and this study represents the most comprehensive application of methods to date in the integrated species delimitation of *Prorocentrum*.

### Evolutionary analysis between the orthologous genes

**Fig 6:**
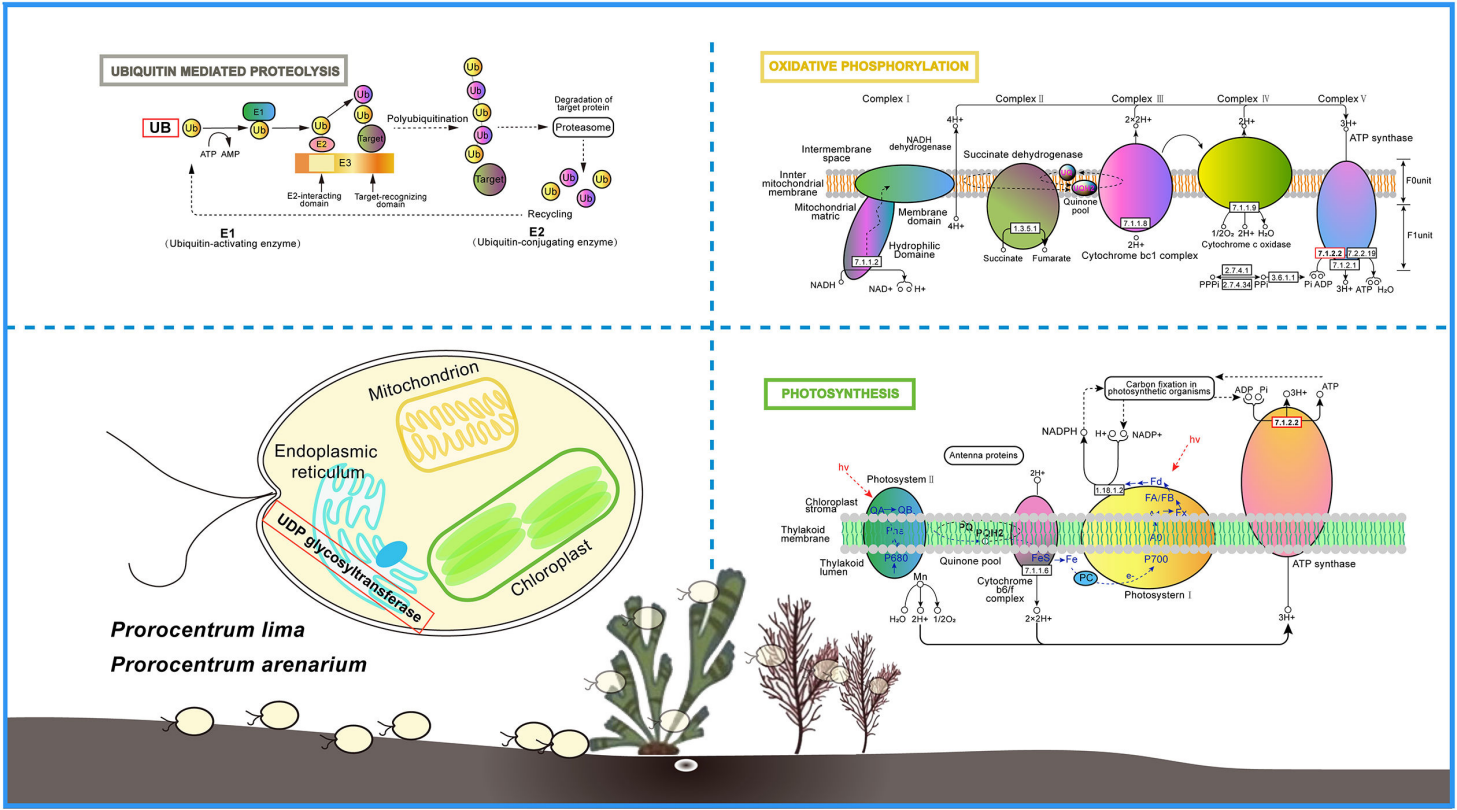
Schematic of adaptation in the evolution of *P. lima and P. arenarium*. A dark red box indicates a positive selection site.

The study identified direct homologous genes that are considered to be conserved and subject to purifying selection, with Ka/Ks values <0.1 (59), including HN341 and XS336 (11,936 genes) (Table S7), as well as HN231 and 3XS36 (12,078 genes) (Table S8). Only a very small number of genes were under strong positive selection. Although some genes under positive selection were identified in the study, Ka/Ks calculations were conservative, and some genes that were under positive selection may not have been detected (60). Candidate positively selected genes may play an important role in species formation and adaptive evolution of *P. lima* and *P. arenarium*. The PSGs for HN341, XS336, and 3XS36 all included UDP-glucosyltransferases (UGTs) (Fig. 5A through D). UGTs (EC 2.4.1.17), also known as glucuronosyltransferases, are pivotal phase II enzymes in the detoxification systems found across all living organisms (61). These enzymes facilitate the conjugation of small lipophilic substances with sugar donors derived from UDP (such as UDP-glucose, UDP-glucuronic acid, UDP-xylose, UDP-galactose, etc.), resulting in the formation of water-soluble products (61). These products can be readily excreted, serving as a protective mechanism to safeguard cellular systems against damage caused by harmful xenobiotics and endogenous compounds (62, 63). These enzymes play a role in several physiological processes such as detoxification, olfaction, cuticle formation, and pigmentation (64). UGTs are associated with detoxification, host adaptation, and insecticide resistance (65). In addition, the expression pattern of detoxification genes can help *P. lima* and *P. arenarium* use a variety of plant hosts, including seagrasses and seaweeds (66–68). The ubiquitin proteolytic system plays a crucial role in various fundamental cellular processes, including cell cycle regulation, modulation of immune and inflammatory responses, control of signal transduction pathways, and development and differentiation (69). In *P. lima* and *P. arenarium*, PSGs related to ubiquitin (UB) participate in the ubiquitin proteolytic system, which is of significant importance for their growth in diverse environments (Fig. 6). F-ATPase, also known as F-Type ATPase, is an enzyme complex found in bacterial plasma membranes, mitochondrial inner membranes, and chloroplast thylakoid membranes (Fig. 6). It utilizes a proton gradient to synthesize ATP by allowing protons to passively move across the membrane, driven by their electrochemical gradient. The energy released during this transport reaction is used to release newly formed ATP from the active site of F-ATPase (70). Positive selection has been observed in F-ATPase genes in *P. lima and P. arenarium*, indicating that the photosynthetic pathway of chloroplasts and the oxidative phosphorylation pathway of mitochondria in benthic diatoms have undergone specific changes during evolution to adapt to benthic habitats (Fig. 6). The diversity of the living environment of *P. lima* and *P. arenarium* leads to more effective selection of cells to counteract harmful factors. This indicates that the process of environmental adaptation is crucial for the evolution of *P. lima* and *P. arenarium*.

Marine viruses have been found to exist in large numbers in all living things, from bacteria to whales in the oceans (71, 72). Viruses can affect the ecology and evolution of their host populations (36). This suggests that viral adhesion in the ocean affects the evolution of *P. lima*, promoting intraspecific diversity. It has been reported that viral genes have been widely endogenized in various green algae (73), which may be the reason for the large genome size of *P. lima*. Combining the isolation, cross-infection of *P. lima* and viral strains may enable an understanding of the unique infection characteristics of *P. lima* and the potential adaptive costs of host resistance, better understanding of *P. lima*-virus interactions has the potential to improve predictive models for algal blooms. In this research, a comprehensive examination of the interspecific boundary, evolution, and distinctions between *P. lima* and *P. arenarium* was conducted. To enable the accurate identification of an autonomous eukaryotic algal species, we developed an evolutionary tree methodology based on orthologous gene sequences and merged the outcomes of ABGD and PTP models. This approach serves as a point of reference for investigating other species with complicated and ambiguous identities and without genomic data, and devising methods for enhancing the taxonomy of microalgae, thereby promoting research on microbial biodiversity.

## MATERIALS AND METHODS

### Algal culture

The Research Center for Harmful Algae and Marine Biology at Jinan University in Guangzhou, China, provided three strains of *Prorocentrum* sp. that produce OA and DTXs and were collected from coastal regions (Table S1). These strains were cultured in L1-Si medium at a temperature of 25°C and a photoperiod of 12-hour light/12-hour dark with an irradiance of 130 µmol·m^–2^·s^–1^. Cultures were incubated for 5, 10, 15, 20, 25, and 30 days. Enumeration of cells, toxin profiling, and toxicity evaluation were conducted every 5 days. The growth rate (μ) was determined by calculating the slope of the natural log of cell counts plotted against culture day during the exponential growth phase. The maximum cell generation time (*G*) was calculated using the formula: *G* = [ln 2]/μ.

### Cell morphology

In this study, morphological characteristics of *Prorocentrum* strains (HN231, XS336, and 3XS36) were studied using light microscopy, fluorescence microscope, and scanning electron microscopy. All cells were fixed in Lugol’s iodine solution and viewed under an Olympus BX61 (Olympus, Tokyo, Japan) during the exponential growth phase of the culture. Photomicrographs were taken with a QImaging Retiga 4000R digital camera (QImaging, Surrey, British Columbia, Canada). Morphological characterization and cell dimensions were determined using IMG Pro plus 6.0 image acquisition and analysis software at ×400 magnification. The length-to-width ratio was calculated, and statistical analysis was performed using SPSS Statistics v22.0 (2015). The L/W ratio variations were evaluated using a one-way ANOVA test (*P*＜0.05). Live cells were taken micrograph at ×400 magnification for the observations of shape and location of chloroplasts. For SEM, after ethanol dehydration, the samples were then dried with CO_2_ at the critical point to prevent cell deformation (CPD 030; Bal-Tec, AG, Balzers, Liechtenstein). The samples were mounted on aluminum stubs, coated with gold in a vacuum sputter coater (EM SCD 500, Leica, Wetzlar, Germany), and examined using a Zeiss Ultras 55 field emission SEM (Zeiss, Jena, Germany).

### Toxin extraction and analysis

Perform toxin analysis according to the method described by Nishimura et al. (44). A crude toxin extract (1 mL) was taken and mixed with 60 µL of 2.5 M NaOH, followed by incubation in a water bath at 70°C for 40 min. After cooling to room temperature, 60 µL of 2.5 M HCl was added to the mixture, which was then filtered through a 0.22-µm spin filter (Pall Corporation, USA) and stored at −20°C for subsequent analysis by liquid chromatography. The reference standards for the three DSTs (OA, DTX1, and DTX2) were procured from the National Research Council of Canada’s Institute for Marine Biosciences, and absolute quantification was performed. Regression curves were constructed based on the known toxin concentrations and spectral areas, enabling the quantification of toxin levels in the samples. Analytical software was utilized to visualize the toxin spectra and facilitate quantification. The determination and quantification of LC/MS were carried out using an HPLC system (Shimadzu Prominence LC-20ADXR) coupled with a tandem mass spectrometer (4500 QTRAP LC-MS/MS system, AB Sciex Instruments, Foster City, CA). Phenomenex Kinetex XB-C18 (150 × 2.1 mm, 2.6 µm) column was utilized for toxin separation, with the mobile phase consisting of acetonitrile (solvent A) and 0.15% formic acid in water (solvent B).

### Phylogenetic and sequence analyses and species delimitation methods

DNA extraction was done using the HP Plant DNA Kit (Omega D2485-01, USA). The entire internal transcribed spacer region was amplified using ITSF and ITSR primers (74). LSU rDNA were amplified using D1R and D3B primers (75, 76). PCR experiments use TaKaRa PCR thermal Cycler Dice TP600 (TaKaRa, Kyoto, Japan), and each sample was sequenced in both directions using ABI 3730XL Bigdye v3.1 hybrid technology (Applied Biosystems, Foster City, California, USA). Pairwise comparison was performed using BioEdit v7.2.5 to generate consensus sequences.

The additional sequences used for constructing the phylogenetic tree were sourced from the NCBI, and their accession numbers can be found in Fig. 2 and 3. Phylogenetic analysis was conducted using maximum likelihood (ML) and neighbor-joining methods in MEGA7 (77). The best DNA substitution model fitting our data for LSU rDNA and ITS region sequences was K2 + I and K2 + G (78). The neighbor-joining algorithm (79) was used to generate the initial tree. The evolutionary distances were computed using the *P*-distance method (80) and are in the units of the number of base differences per site. Bayesian inference (BI) was performed using MrBayes v3.1.2 with the SYM + G model as the best-fitting substitution model for both LSU rDNA and the ITS region. Genetic distances of *Prorocentrum* ITS and LSU were estimated using the *P*-distance method in MEGA7 (77).

To distinguish between species in a data array, two species delimitation algorithms were applied to ITS alignments. ABGD uses a method of automatic search for interspecific distance gaps in genetic distance and is available as an online server at https://bioinfo.mnhn.fr. For the ABGD analysis, we employed a matrix of genetic distances calculated using the maximum likelihood method. In our analysis using the ABGD method, we evaluated the results both in the initial partition mode and the recursive partition mode. mPTP analysis was conducted using the PTP model under maximum likelihood estimation (using the -single ML option) and the mPTP model (using the -multi-ML option) using the web service provided at http://mPTP.h-its.org. The phylogenetic tree constructed in the MrBayes program was used to distinguish species using the PTP algorithm, which is available as an online server at https://species.h-its.org/. In our study, we utilized the maximum likelihood method implemented in the MEGA program to construct a phylogenetic tree. This tree was subsequently employed for species differentiation through the PTP algorithm. Furthermore, we employed the ML version of the PTP method, known as bPTP, in conjunction with our phylogeny, using Bayesian analysis.

### RNA extraction

To extract RNA, the cultures (HN231, XS336, 3XS36) were harvested together, immediately frozen in liquid nitrogen, and then stored at −80°C until the RNA extraction process. Each sample was mixed with 1 mL of RNAiso reagent (Takara, China), and RNA was extracted using phenol-chloroform-isopropanol and precipitated with ethanol. The sample concentration and purity were determined using a NanoDropOne (Thermo Scientific, USA). Three biological replicates were set up for each sample and sent to BGI Genomics in Shenzhen for transcriptome sequencing.

### Bioinformatics analysis

The relative expression levels of genes were calculated using log2 (YH29/WH10). Genes with a fold change of more than two and a *Q*-value (adjusted *P*-value) of ≤0.05 were considered significantly differentially expressed. The functional annotation of genes was accomplished by mapping them to various databases (NT, NR, KOG, KEGG) using BLAST software (v2.2.23). The significance levels of terms and pathways were adjusted using a strict threshold of *Q*-value <0.05.

### Orthologous gene analysis

To obtain pairs of sequences with the best hit to each other, the transcriptome sequences of HN231/XS336 and 3XS36 were blasted reciprocally, retaining those with an *E*-value of 1.0E−5.Multiple sequence alignments of the protein sequences from 27,632 orthologous genes were conducted using the MUSCLE software (81), available at http://www.drive5.com/muscle. The NJ tree was then constructed with MEGA, and the Bootstrap method was used to test 1,000 times to generate the final evolutionary trees.

### Ka/Ks analysis of orthologous genes

This study calculated the ratio of the number of nonsynonymous substitutions per nonsynonymous site (Ka) to the number of synonymous substitutions per synonymous site (Ks) to assess the evolutionary pressure on the genes analyzed (82). Ka/Ks are commonly used to aid in understanding the direction of evolution and its selective strength in a coding sequence (83). The Ka, Ks, and Ka/Ks ratio were computed using paml-codeml. To ensure the reliability of the analysis, genes with dS >2 or NdN <1 or SdS <1, which typically have short differentiation times between closely related species and a low ratio of synonymous substitutions, were removed prior to analysis. We identified that orthologous genes with a Ka/Ks ratio greater than 1 were under strong positive selection. Orthologous genes with a Ka/Ks ratio between 0.5 and 1 were under weak positive selection, while those with a Ka/Ks ratio below 0.1 were under negative selection (purifying selection).

## Data Availability

The RNA-seq data of this study have been submitted to NCBI under accession no. PRJNA1051965.

## SUPPLEMENTAL MATERIAL

Fig. S1 spectrum.02367-23-s0001.tif Mean L/W ratio among three strains.

Fig. S2 spectrum.02367-23-s0002.tif Growth curve and cell growth rates over time for the cultivated strains.

Fig. S3 spectrum.02367-23-s0003.tif Free and total intracellular OA, DTX1 and DTX2 isomer toxin concentrations and productivity in the cultured strains.

Fig. S4 spectrum.02367-23-s0004.tif Scatter plot of concentrations of free/total OA and DTX1 in XS336, as well as free/total OA and DTX2 isomer in 3XS36.

Fig. S5 spectrum.02367-23-s0005.tif Gene ontology (GO) annotation of strong positive selection genes between strains.

Supplemental legends spectrum.02367-23-s0006.docx Legends for supplemental figures and tables.

Supplemental tables spectrum.02367-23-s0007.xlsx Tables S1 to S9.

ASM does not own the copyrights to Supplemental Material that may be linked to, or accessed through, an article. The authors have granted ASM a non-exclusive, world-wide license to publish the Supplemental Material files. Please contact the corresponding author directly for reuse.
