# Supplementary figures and images for: Refining taxonomic identification of microalgae through molecular and genetic evolution: a case study of Prorocentrum lima and Prorocentrum arenarium
Source: Microbiol Spectr. 2024 Apr 4;12(5):e02367-23. doi: 10.1128/spectrum.02367-23 (PMC11064606; doi:10.1128/spectrum.02367-23)

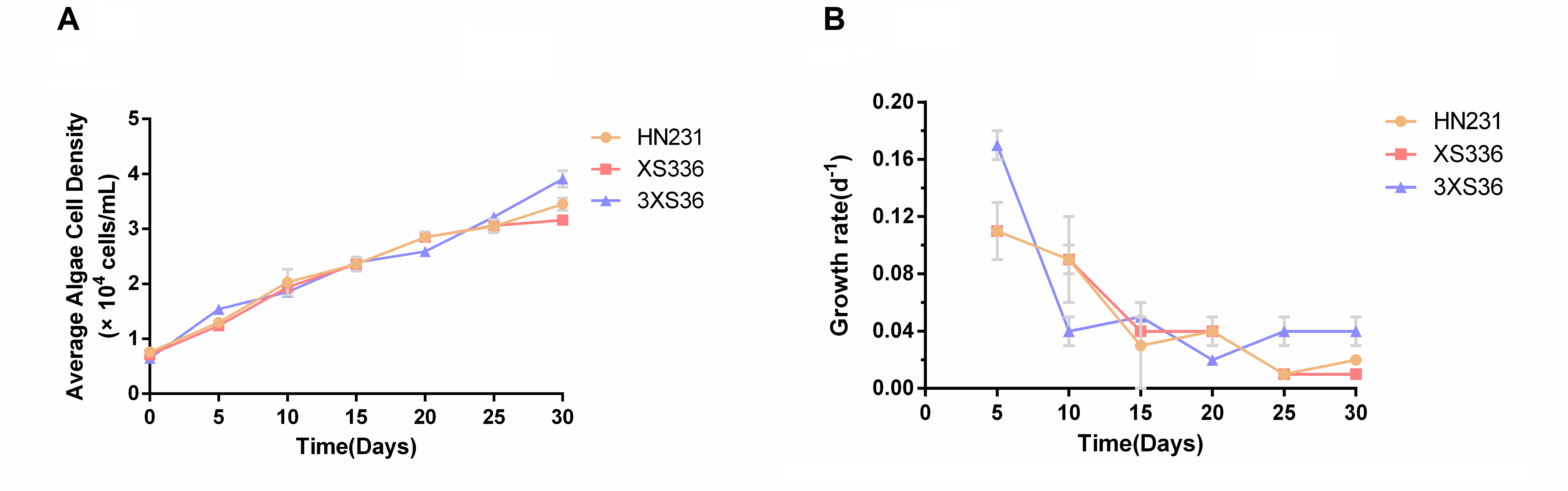

Supplement: Fig. S2 — Growth curve and cell growth rates over time for the cultivated strains. [file spectrum.02367-23-s0002.tif]

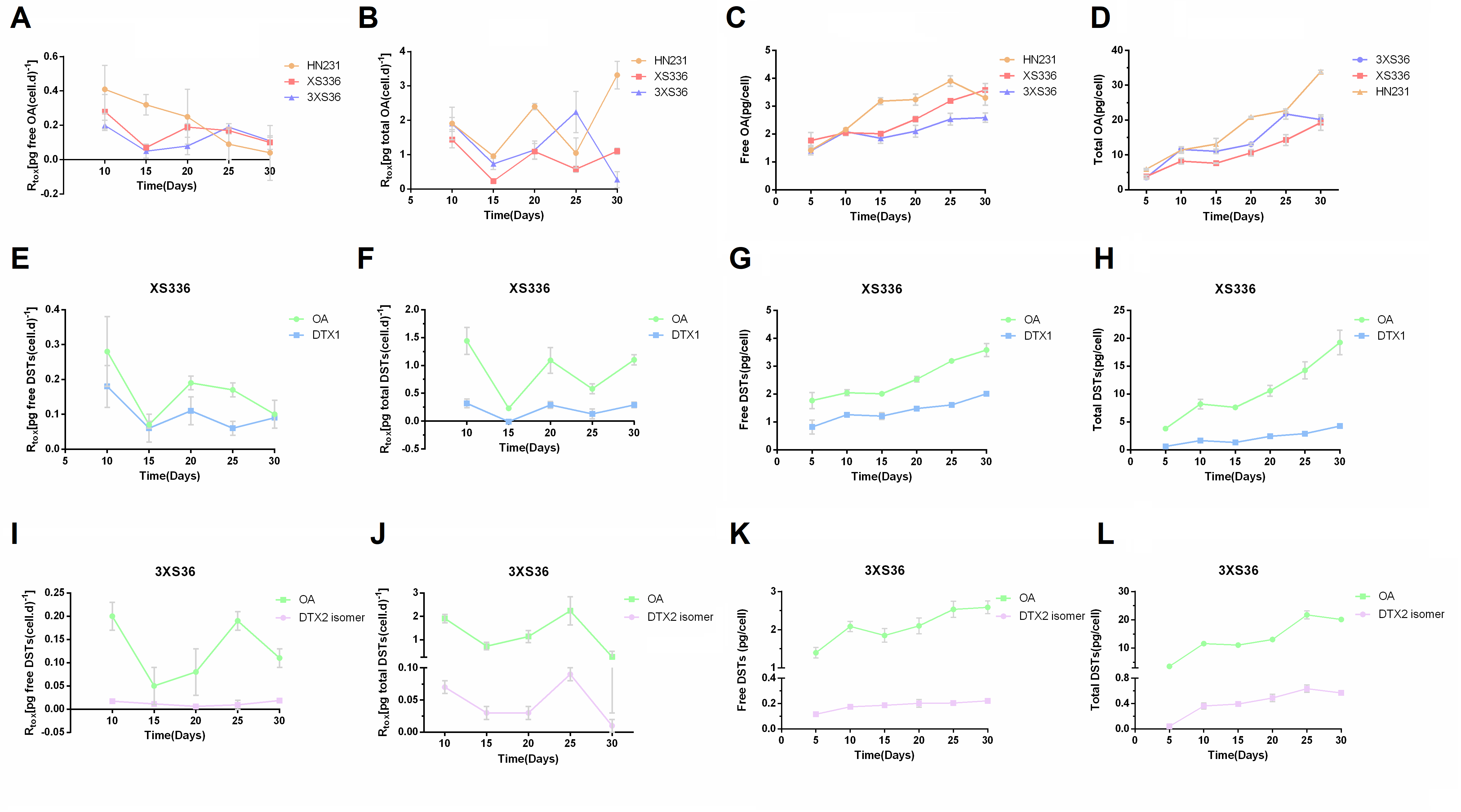

Supplement: Fig. S3 — Free and total intracellular OA, DTX1 and DTX2 isomer toxin concentrations and productivity in the cultured strains. [file spectrum.02367-23-s0003.tif]

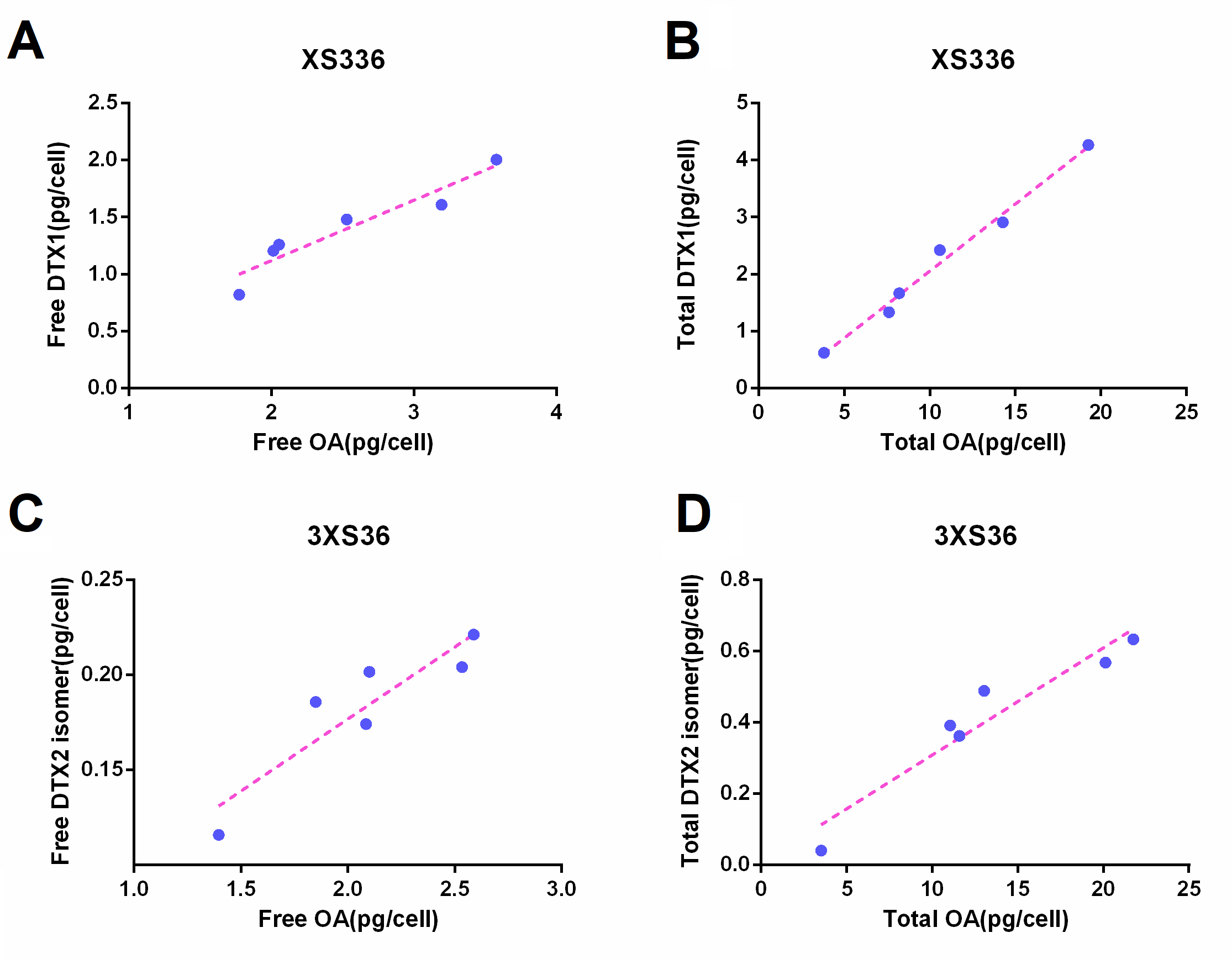

Supplement: Fig. S4 — Scatter plot of concentrations of free/total OA and DTX1 in XS336, as well as free/total OA and DTX2 isomer in 3XS36. [file spectrum.02367-23-s0004.tif]

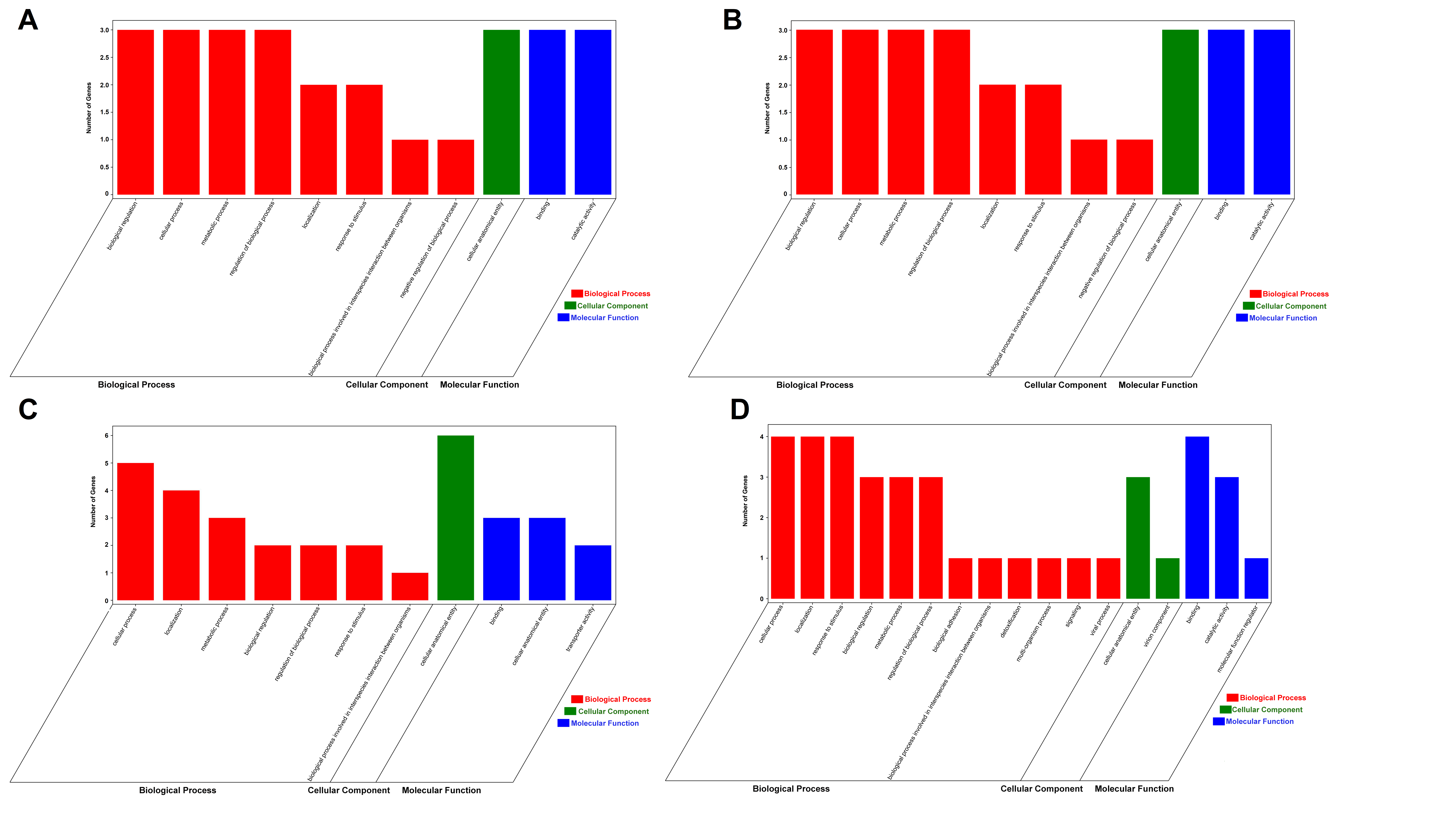

Supplement: Fig. S5 — Gene ontology (GO) annotation of strong positive selection genes between strains. [file spectrum.02367-23-s0005.tif]
