# Supplementary material for: Refining taxonomic identification of microalgae through molecular and genetic evolution: a case study of Prorocentrum lima and Prorocentrum arenarium
Source: Microbiol Spectr. 2024 Apr 4;12(5):e02367-23. doi: 10.1128/spectrum.02367-23 (PMC11064606; doi:10.1128/spectrum.02367-23)
Supplement: Supplemental legends — Legends for supplemental figures and tables. [file spectrum.02367-23-s0006.docx]

**Fig S1** Mean L/W ratio among three strains (HN231 N=138; XS336 N=101; 3XS36 N=91). The bars represent standard deviation of duplicate measurements. Letters on the top of columns show statistically significant differences observed with a one-way ANOVA test (P＜0.05).

**Fig S2** The graph shows (A) the growth curve and (B) cell growth rates over time for the cultivated strains. Each data point represents the mean of three measurements, and the error bars indicate the range of one standard deviation.

**Fig S3** Free and total intracellular OA, DTX1 and DTX2 isomer toxin concentrations and productivity in the cultured strains. Productivity of (A) free OA and (B) total in the cultured strains (HN231, XS336 and 3XS36); concentrations of (C) free OA and (D) total in the cultured strains (HN231, XS336 and 3XS36); productivity of (E) free / (F) total OA and DTX1 in the XS336 strain; concentrations of (G) free / (H) total OA and DTX1 in the XS336 strain; productivity of (I) free / (J) total OA and DTX2 isomer in the 3XS36 strain; concentrations of (K) free / (L) total OA and DTX2 isomer in the 3XS36 strain. Each data point represents the mean of three measurements, and the error bars indicate the range of one standard deviation.

**Fig S4** Scatter plot of concentrations of (A) free / (B) total OA and DTX1 in the XS336; scatter plot of concentrations of (C) free / (D) total OA and DTX2 isomer in the 3XS36.

**Fig S5** Gene ontology (GO) annotation of strong positive selection genes between strains. HN231 (A) VS XS336 (B); HN231 (C) VS 3XS36 (D). The right y-axis represents the number of genes annotated to a certain GO term, and the x-axis represents the detailed classification of each GO term.

**Table S1**. Benthic *Prorocentrum* observed in this study.

**Table S2**. Summary of Initial cell density, Max. cell density, Average cell growth rates, Max. cell growth rates and Average cell generation time in the cultured strains.

**Table S3**. Free and total intracellular OA, DTX1 and OA isomer toxin concentrations and productivity in the cultured strains.

**Table S4**. Estimation of average uncorrected p-distances of the LSU rDNA (970 positions) calculated among *P. lima* comple, *P. arenarium*, *P. porosum*, *P.* cf. *lima*, *P. caipirignum*, *P. hoffmannianum*, *P. mexicanum* and *P. rhathymum*. Data are average (max-min) uncorrected p distances for within species/species complex/phylotype/subclade and among them.

**Table S5**. Estimation of average uncorrected p-distances of the ITS rDNA (656 positions) calculated among *P. lima* comple, *P. arenarium*, *P. porosum*, *P.* cf. *lima*, *P. caipirignum* and *P. hoffmannianum*. Data are average (max-min) uncorrected p distances for within species/species complex/phylotype/subclade and among them.

**Table S6**. List of single-copy orthologous genes between strains.

**Table S7**. Ka/Ks analysis of orthologous genes between HN231 and XS336.

**Table S8**. Ka/Ks analysis of orthologous genes between HN231 and 3XS36.

**Table S9**. Abbreviation vocabulary summary in the article.
